# Enhancing the catalytic activity of a novel GH5 cellulase *Gt*Cel5 from *Gloeophyllum trabeum* CBS 900.73 by site-directed mutagenesis on loop 6

**DOI:** 10.1186/s13068-018-1080-5

**Published:** 2018-03-22

**Authors:** Fei Zheng, Tao Tu, Xiaoyu Wang, Yuan Wang, Rui Ma, Xiaoyun Su, Xiangming Xie, Bin Yao, Huiying Luo

**Affiliations:** 1 Key Laboratory for Feed Biotechnology of the Ministry of Agriculture, Feed Research Institute, Chinese Academy of Agricultural Sciences, No. 12 South Zhongguancun Street, Haidian, Beijing, 100081 People’s Republic of China; 2 College of Biological Sciences and Biotechnology, Beijing Forestry University, Beijing, 100083 People’s Republic of China

**Keywords:** GH5 cellulase, Saturation mutation, Catalytic activity, Loop region

## Abstract

**Background:**

Cellulases of glycosyl hydrolase (GH) family 5 share a (β/α)_8_ TIM-barrel fold structure with eight βα loops surrounding the catalytic pocket. These loops exposed on the surface play a vital role in protein functions, primarily due to the interactions of some key amino acids with solvent and ligand molecules. It has been reported that motions of these loops facilitate substrate access and product release, and loops 6 and 7 located at the substrate entrance of the binding pocket promote proton transfer reaction at the catalytic site motions. However, the role of these flexible loops in catalysis of GH5 cellulase remains to be explored.

**Results:**

In the present study, an acidic, mesophilic GH5 cellulase (with optimal activity at pH 4.0 and 70 °C), *Gt*Cel5, was identified in *Gloeophyllum trabeum* CBS 900.73. The specific activities of *Gt*Cel5 toward CMC-Na, barley β-glucan, and lichenan were 1117 ± 43, 6257 ± 26 and 5318 ± 54 U/mg, respectively. Multiple sequence alignment indicates that one amino acid residue at position 233 on the loop 6 shows semi-conservativeness and might contribute to the great catalytic performance. Saturation mutagenesis at position 233 was then conducted to reveal the vital roles of this position in enzyme properties. In comparison to the wild type, variants N233A and N233G showed decreased optimal temperature (− 10 °C) but increased activities (27 and 70%) and catalytic efficiencies (*k*_cat_/*K*_m_; 45 and 52%), respectively. The similar roles of position 233 in catalytic performance were also verified in the other two GH5 homologs, *Te*Egl5A and *Po*Cel5, by reverse mutation. Further molecular dynamics simulations suggested that the substitution of asparagine with alanine or glycine may introduce more hydrogen bonds, increase the flexibility of loop 6, enhance the interactions between enzyme and substrate, and thus improve the substrate affinity and catalytic efficiency.

**Conclusion:**

This study proposed a novel cellulase with potentials for industrial application. A specific position was identified to play key roles in cellulase–substrate interactions and enzyme catalysis. It is of great importance for understanding the binding mechanism of GH5 cellulases, and provides an effective strategy to improve the catalytic performance of cellulases.

**Electronic supplementary material:**

The online version of this article (10.1186/s13068-018-1080-5) contains supplementary material, which is available to authorized users.

## Background

Lignocellulose is composed of cellulose, hemicellulose, and lignin, and represents the most abundant renewable carbon source on earth [1]. The enzymatic hydrolysis of polysaccharides to monosaccharides is crucial from both viewpoints of cost and efficiency in the current practice of converting lignocellulosic biomass into biofuel. Complete hydrolysis of cellulose requires the cooperative actions of three types of cellulases: endoglucanase (EC 3.2.1.4) that randomly cleaves the internal β-1,4-glycosidic bonds; cellobiohydrolase (exoglucanase; EC 3.2.1.91) that processively acts on the chain termini to release cellobiose; and β-glucosidase (EC 3.2.1.21) that hydrolyzes cellobiose to glucose [2].

Based on the sequence and structure similarity of CAZymes (http://www.cazy.org), endoglucanases are grouped into 13 glycoside hydrolase (GH) families, including GH5-9, 12, 44, 45, 48, 51, 74, 124, and 131 [3]. Of these, GH5 is the largest and the most functionally diverse group, and those from fungi are mainly confined into subfamily GH5_5 with *endo*-β-1,4-glucanase activity [4]. So far, six eukaryotic GH5 endoglucanases from *Piromyces rhizinflata* (*Pr*EglA) [5], *Thermoascus aurantiacus* (*Ta*Cel5A) [6], *Hypocrea jecorina* (*Trichoderma reesei*) (*Tr*Cel5A) [7], *Ganoderma lucidum* (*Gl*Cel5A) [8], *Aspergillus niger* (*An*Cel5A) [9], and *Penicillium verruculosum* (PDB No. 5I6S) have been resolved. The typical catalytic domain of a GH5 cellulase has a canonical (β/α)_8_ TIM-barrel fold, in which the eight parallel β-strands and eight α-helices are connected by seven βα or αβ loops [10].

The loops that connect secondary structures are frequently located on the protein surface and are critical for substrate specificity and catalytic activity. For example, the mutation T113R of polygalacturonase PG8fn increased the plasticity of T3 loop and caused an improvement of the catalytic efficiency by ~ 2.4-fold [11]; modifying the loop conformations of two GH6 cellobiohydrolases facilitated the cellulose chain gliding and allowed more occasional endo-cleavages [12, 13]; and deletion of an exo-loop of a bacterial cellobiohydrolase altered its endolytic activity [14]. A few studies also reported the effects of loops on cellulases. For the cellulase Cel12A from *Thermotoga maritima*, a Tyr-to-Gly mutation on a unique loop related to substrate binding led to an increased specific activity by 1.7-fold [15]. The protonation state of the catalytic glutamates of Cel5B from *Clostridium thermocellum*, with or without substrate, is largely governed by the conformational changes of β_3_α_3_ loop [16]. When replacing the Phe267 with Ala of cellulase *Gt*Cel5E from *Clostridium thermocellum*, its hydrophobic interactions with two other residues were broken, the flexible loop was relocated, and the variant displayed an increased *k*_cat_ value by fourfold [17]. These previous studies altogether reveal the importance of loop structures in enzyme catalysis.

Protein engineering is a prevalent method with numerous successes for enzyme improvements [18, 19]. Site-directed mutagenesis based on rational design has been widely used to identify the roles of a specific amino acid residue. In the present study, a novel cellulase of GH5 from *Gloeophyllum trabeum* CBS 900.73, designated *Gt*Cel5, was produced in *Pichia pastoris* GS115. *Gt*Cel5 with great catalytic performance had asparagine at position 233 of loop 6 (βα loop), the same as the structure-resolved homologs *Gl*Cel5A (72%, 5D8W) and *Tr*Cel5A (69%, 3QR3) of GH5_5 [3, 4]. In contrast, some other GH5 cellulases have glycine at this position. In order to gain insights into the functional role of loop 6 in GH5 cellulases, we created saturation mutants of *Gt*Cel5 at position 233 by site-directed mutagenesis. The results were then verified by reverse mutation on two GH5 homologs. Biochemical and bioinformatics analyses indicated that residue 233 on loop 6 is critical for the substrate binding and catalytic efficiency.

## Methods

### Strains and plasmids

The donor strain *G. trabeum* CBS 900.73 from the CBS-KNAW Fungal Biodiversity Center (Utrecht, the Netherlands) was grown at 30 °C for 3 days in a lignocellulose medium containing (w/v) 5 g/L NaCl, 5 g/L (NH_4_)_2_SO_4_, 1 g/L KH_2_PO_4_, 0.5 g/L MgSO_4_·7H_2_O, 0.2 g/L CaCl_2_, 0.01 g/L FeSO_4_·7H_2_O, 15 g/L corncob, 15 g/L soybean meal, and 15 g/L wheat bran. Plasmid pPIC9 harboring an ampicillin resistance gene was used for selection in *Escherichia coli* Trans I-T1 (TransGen, Beijing, China). Transformed *E. coli* was maintained on LB medium supplemented with 100 μg/mL ampicillin and grown at 37 °C. Cellulases *Te*Egl5A from *Talaromyces emersonii* [20] and *Po*Cel5 from *Prosthecium opalus* (unpublished data) were selected for reverse mutation. Plasmids pPIC9-*Teegl5A* and pPIC9-*Pocel5* containing the cDNA fragments of mature *Te*Egl5A and *Po*Cel5-encoding sequences were used as the PCR templates. Plasmid DNA was isolated from *E. coli* using a Qiagen Miniprep Kit. *P. pastoris* GS115 (Invitrogen) was maintained on MD plates (2% glucose and 2% agarose) at 30 °C.

### Gene cloning

Fungal RNA was isolated and purified from 100 mg of 3-day-old mycelia grown in the lignocellulose medium using the SV Total RNA Isolation System (Promega). cDNAs were synthesized in vitro using the ReverTra Ace-a-TM kit (TOYOBO, Osaka, Japan) with total RNA as the template. Amplification of the *Gt*Cel5 gene was carried out using the oligonucleotide primer set *Gt*Cel5-F and *Gt*Cel5-R (5′-CCGAATTCGCCGCGCTCTCTCCGAGAGTGACA-3′, and 5′-ACTGCGGCCGCTCATGCGTTGGCAATCGGAGCCAAGCA-3′, with the *Eco*RI and *Not*I restriction sites underlined). The 50-μL PCR contained 10 μg of cDNA template, 5 μM of each primer, 1 mM of dNTPs, 5 μL of 10× PCR buffer, and 1 μL of Taq DNA polymerase (Fermentas; 2.5 U/μL). The specific PCR products were digested with *Eco*RI and *Not*I to create sticky ends and ligated to the *Eco*RI–*Not*I-digested vector pPIC9 using T4 DNA ligase (New England Laboratory). The constructed recombinant plasmid pPIC9-*Gtcel5* was then transformed into *E. coli* Trans I-T1 competent cells. Positive transformants were sequenced for verification.

### Sequence analysis

The DNA and amino acid sequences were analyzed using the BLASTx and BLASTp programs (http://www.ncbi.nlm.nih.gov/BLAST/), respectively. The introns, exons, and transcription initiation sites were predicted using the GENSCAN Web Server (http://genes.mit.edu/GENSCAN.html). SignalP 3.0 was used to predict the signal peptide sequence (http://www.cbs.dtu.dk/services/SignalP/). The potential *N*-glycosylation sites were predicted online (http://www.cbs.dtu.dk/services/NetNGlyc/). Sequence assembly and estimation of the molecular mass and *p*I of the mature peptide were performed using the Vector NTI Suite 10.0 software (Invitrogen). MEGA 4.0 was used for inferring the phylogenetic relationship of GH5 cellulases [21].

### Selection of the mutation site and site-directed mutagenesis

**Fig. 1:**
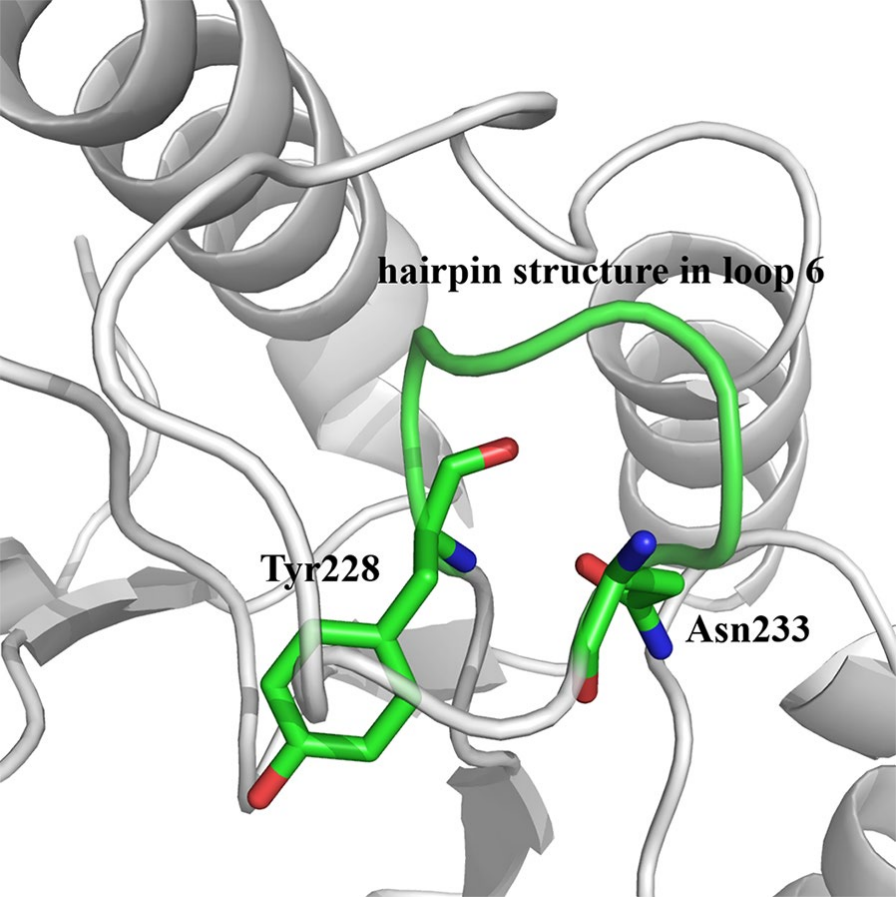
Modeled structure of *Gt*Cel5. The unique hairpin structure is shown in green. Residues Tyr228 and Asn233 involved in the movement loop 6 are indicated

Loop 6 of *Gt*Cel5 is in close proximity to the catalytic pocket and the component residues YLDSDN are capable of forming a unique hairpin structure (Fig. 1). Identification and multiple sequence alignment of 51 fungal cellulases of GH5 were conducted using the FASTA [22] and ClustalW [23] algorithms. Based on the structure and sequence analysis of loop 6, a key residue probably related to *Gt*Cel5 functionality was identified, and selected for saturation mutagenesis. With recombinant plasmids pPIC9-*Gtcel5*, pPIC9-*Teegl5A*, and pPIC9-*Pocel5* as the templates, the mutants were first constructed by overlap PCR for preliminary screening. Reverse mutations of G216A and G216N of *Te*Egl5A and G210A and G210N of *Po*Cel5 were performed using the Fast Mutagenesis System Kit (TransGen) with 30 amplification cycles. The primer pairs used in this study are listed in Additional file 1: Table S1.

### Enzyme expression and purification

Recombinant plasmids containing the gene fragments coding for the wild-type and mutant enzymes of *Gt*Cel5, *Te*Egl5A, and *Po*Cel5 were then linearized with *Bgl*II for transformation into *P. pastoris* GS115. The positive transformants were screened on MD plates. Ninety-six positive transformants of each enzyme were selected to grow in 3 mL BMGY at 30 °C for 48 h, collected, and resuspended in 1 mL BMMY containing 0.5% methanol for 72-h enzyme induction at 30 °C. The culture supernatants of each transformant were collected by centrifugation at 12,000×*g* for 10 min at 4 °C and examined by activity assay. The transformants showing the highest cellulase activities were inoculated into 30 mL YPD and incubated at 30 °C, 200 rpm for 48 h, and transferred into 400 mL BMGY in 1-L Erlenmeyer flasks for 48-h growth. Cells were then harvested by centrifugation at 4500×*g* for 5 min at 4 °C and resuspended in 200 mL of BMMY containing 0.5% (v/v) methanol for 48 h at 30 °C for induction.

Cell-free cultures were collected by centrifugation at 12,000×*g* for 10 min at 4 °C. Further purification was performed using the HiTrap Q HP anion exchange column (Amersham Biosciences, Uppsala, Sweden). Binding buffer was composed of 10 mM sodium phosphate (pH 7.5). Elution was performed using a linear gradient of 0–1 M sodium chloride in the same buffer. The purities of the enzymes were checked with 12% SDS–polyacrylamide gel electrophoresis (PAGE) and Coomassie blue staining. *Endo*-β-*N*-acetylglucosaminidase H (*Endo* H) from New England Biolabs was used to remove *N*-glycosylation according to the manufacturer’s instructions. Purified proteins were quantified using the Bradford protein assay kit (Bio-Rad) and then used for enzyme characterization.

### Cellulase activity assay

CMC-Na (medium viscosity) from Sigma-Aldrich at a concentration of 10 mg/mL was used as the substrate. The assay mixtures contained 900 μL of substrate solution in 100 mM McIlvaine buffer (optimal pH) and 100 μL of appropriately diluted enzyme. The reaction mixtures were incubated at optimal temperature for 10 min, followed by the addition of 1.5 mL 3,5-dinitrosalicylic acid (DNS) and incubation in a 100 °C water-bath for 5 min [24]. The amounts of reducing sugar released were measured at 540 nm, and one unit of the cellulase activity was defined as the amount of enzyme that released 1 μmol of reducing sugar per minute.

### Biochemical characterization

CMC-Na was used as the substrate for enzyme characterization. The buffers used were 100 mM KCl–HCl (pH 1.0–3.0), 100 mM citric acid–Na_2_HPO_4_ (pH 2.2–7.0), 100 mM Tris–HCl (pH 8.0–9.0), and 100 mM glycine–NaOH (pH 9.0–12.0). The pH–activity profile of each enzyme was determined at optimal temperature in buffers of pH 2.2–8.0. The temperature–activity profile of each enzyme was determined at optimal pH over the temperature range from 40 to 90 °C. For pH stability, each enzyme was preincubated at 37 °C for 1 h in buffers of different pH (1.0–12.0) and subjected to the residual activity assay under standard conditions as described above. For thermostability assay, each enzyme (approximately 100 μg/mL) was preincubated at 60 or 70 °C for 0–60 min, and aliquots of 100 μL were withdrawn at specific time points for residual activity assay.

### Substrate specificity

Polysaccharides from Sigma-Aldrich and Megazymes (Wicklow, Ireland) containing different glycosidic linkages, including CMC-Na, barley β-glucan, lichenan, laminarin, konjac glucomannan, Avicel, locust bean gum, xylan, and filter paper, were used to test the substrate specificity of *Gt*Cel5 under standard conditions. The specific activities of *Gt*Cel5 variants toward barley β-glucan and CMC-Na were also determined and compared to that of the wild type.

### Kinetic assays

Kinetic parameters of the enzymes were derived from the reactions under optimal conditions with 0.125–10 mg/mL CMC-Na as the substrate. Initial velocities were determined by measuring the production rates of reducing sugar with the DNS method. The kinetic parameters (apparent *K*_m_ and *k*_cat_) were calculated using the GraphPad Prism 6.0 (http://www.graphpad.com/scientific-software/prism/) and the nonlinear regression algorithm embedded in the enzyme kinetics module. The catalytic efficiency (*k*_cat_/*K*_m_) of each enzyme was then calculated.

### Bioinformatic analyses

Discovery Studio 2017 software was used for automated comparative modeling of *Gt*Cel5 and its variants with *Tr*Cel5A (3QR3, 69% identity) as the template. To explore the possible roles of site-directed mutagenesis at position 233, molecular dynamic (MD) simulation was conducted to compare the dynamic properties of monomeric *Gt*Cel5 and its variants N233A, N233D, and N233G. All of the MD simulations were carried out using the Amber 14 package at a temperature of 323K for 20 ns. Force field ff99SB with the TIP3P water model was used to describe the systems [25–27]. All protein atoms were at least 12 Ȧcc from the edge of the water box. The systems had net negative charges and were neutralized by addition of sodium ions with the Amber tool program [28]. Prior to the MD simulations, each system was carried out with 10,000 steps of steepest descent for energy minimizations. The trajectories of the first 5 ns were treated as equilibration periods, and the trajectories of the last 15 ns were used for data analyses. The root-mean-square deviation (RMSD) and root-mean-square fluctuation (RMSF) values of the C_α_ atoms calculated from the equilibrium state were plotted as a function of residue number.

To analyze the interactions between enzyme and substrate, cellotetraose (G4) was docked to *Gt*Cel5 and its variants N233A and N233G, respectively, using YASARA software (http://www.yasara.org). MD simulations of the enzyme–cellotetraose complex were then carried out at a temperature of 323K for 20 ns. The Amber force fields ff99SB and GLYCAM_06 [26, 29] were employed to model the cellulase and cellotetraose, respectively. Five thousand snapshots taken from the last 5-ns MD trajectories were used for molecular mechanics/Poisson Boltzmann and surface area continuum solvation (MM/PBSA) calculations. The binding free energy between ligand and protein was calculated by the Amber14. The *ΔG* value was determined according to the equation: *ΔG* = *G*_complex_ – *G*_receptor_ – *G*_ligand_. The contributions of internal, electrostatic, and van der Waals’ energy to *ΔG* were analyzed using the force field (http://ambermd.org/tutorials/advanced/tutorial3/py_script/section2.htm) [30].

Hydrogen bond is one of the most important directional intermolecular interactions [31]. Putative hydrogen bonds were assigned based on two geometric criteria: the distance of less than 3.5 Å and the angle larger than 120° between the acceptor and the hydrogen donor. Visualization and figure preparation of the three dimensional molecules were performed using the PyMOL version 1.7.2.1 (Delano Scientific).

## Results

### Cloning and sequence analysis of *Gtcel5*

A GH5 cellulase-encoding gene, *Gtcel5* (GenBank Accession No. XP_007867902), was identified in the genome of *G. trabeum* CBS 900.73. The *Gtcel5* contains 1415 base pair that is composed of 7 exons and 6 introns. Deduced *Gt*Cel5 contained 359 amino acid residues including a putative N-terminal signal peptide of 20 amino acids. The catalytic domain showed the highest amino acid sequence identity of 73% with the Cel4 from *Polyporus arcularius* and the *endo*-β-1,4-glucanase from *Sporotrichum thermophile*, and 72% with the structure-resolved *Gl*Cel5A (5D8W) [8] and 69% with the structure-resolved *Tr*Cel5A (3QR3) [7]. As most fungal cellulases of GH5 (EC3.2.1.4) are classified into subfamily GH5_5 [4], *Gt*Cel5 is closely related to cellulases 5D8W and BAF75943 belonging to the same subfamily (see Additional file 1: Fig. S1). Homology modeling indicated that *Gt*Cel5 folds into a typical (β/α)_8_ structure and contains eight highly conserved residues of GH5 enzymes, including Arg67, His111, Asn154, Glu155, His226, Tyr228, Glu267, and Trp300 (numbering without the signal peptide sequence).

### Selection of the mutagenesis site in *Gt*Cel5

**Fig. 2:**
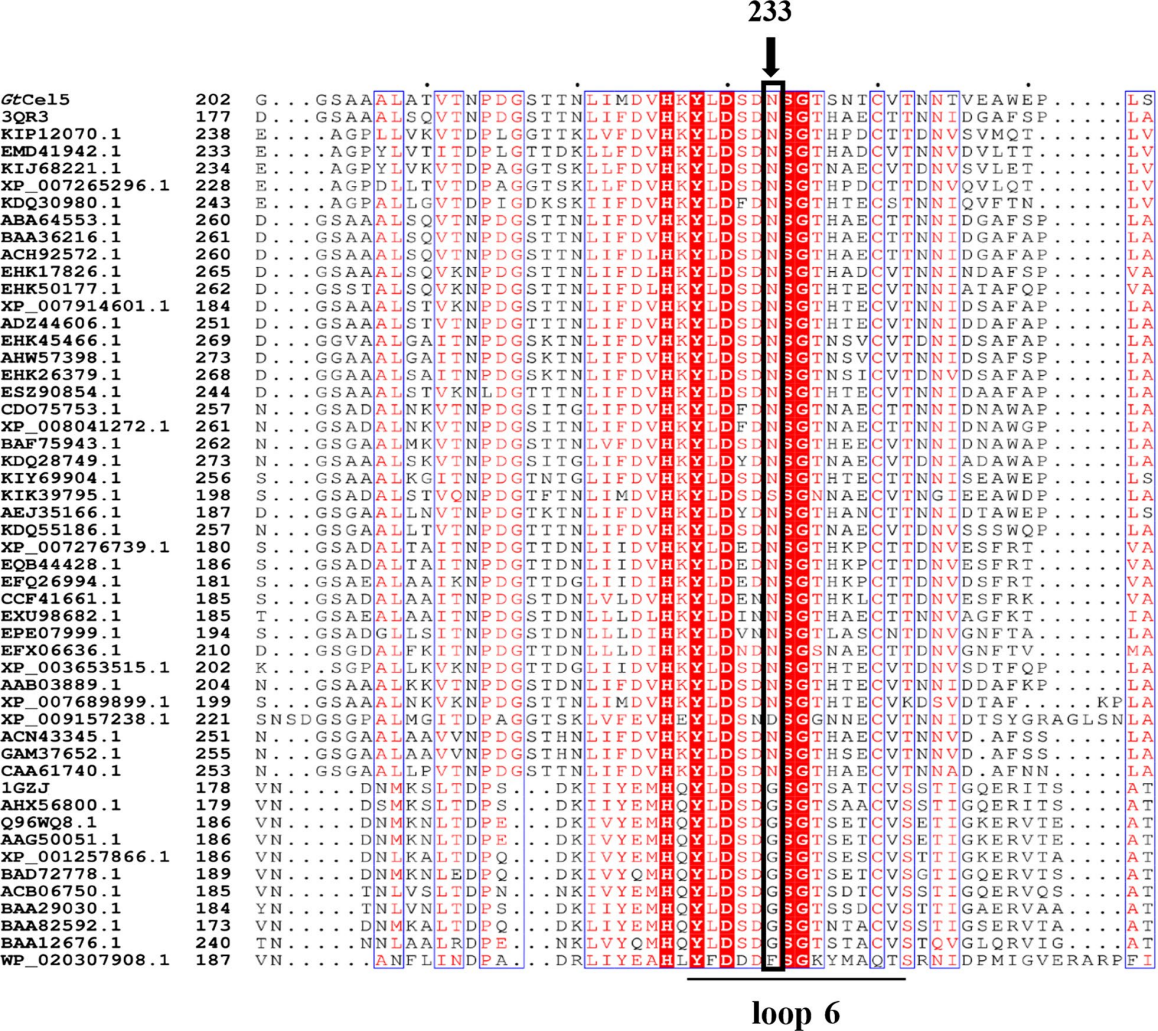
Multiple sequence alignments of 51 fungal cellulases of GH5. Two main groups are classified based on the residue at position 233

Loop regions are proposed to play vital roles in the interactions between TIM-barrel enzymes and substrate [11, 13, 32, 33]. Structure and sequence analysis (Figs. 1, 2) indicated that the Tyr228 and Asn233 of *Gt*Cel5 might be the key switch residues to control the movement of loop 6. The conformational plasticity of this hairpin structure might affect the catalytic performance of *Gt*Cel5. Tyr228 was highly conserved, while Asn233 showed variation. Therefore, we selected N233 for saturation mutagenesis to investigate the effects of the residue at position 233 on catalytic efficiency of GH5 cellulases.

### Production of *Gt*Cel5 and its mutants in *P. pastoris*

*Gt*Cel5 and its 19 mutant enzymes were successfully expressed in *P. pastoris* GS115. One protein band of *Gt*Cel5 with the apparent molecular weight of approximately 40 kDa was detected on the SDS-PAGE (Additional file 2: Fig. S2), which was higher than the calculated value (35.7 kDa). After *Endo* H digestion, the *N*-deglycosylated *Gt*Cel5 decreased to approximately 36 kDa. Mutant enzymes had similar apparent molecular weights, and showed a single band with expected molecular mass after *Endo* H treatment (data not shown).

### Comparison of the enzymatic properties between *Gt*Cel5 and its variants

**Fig. 3:**
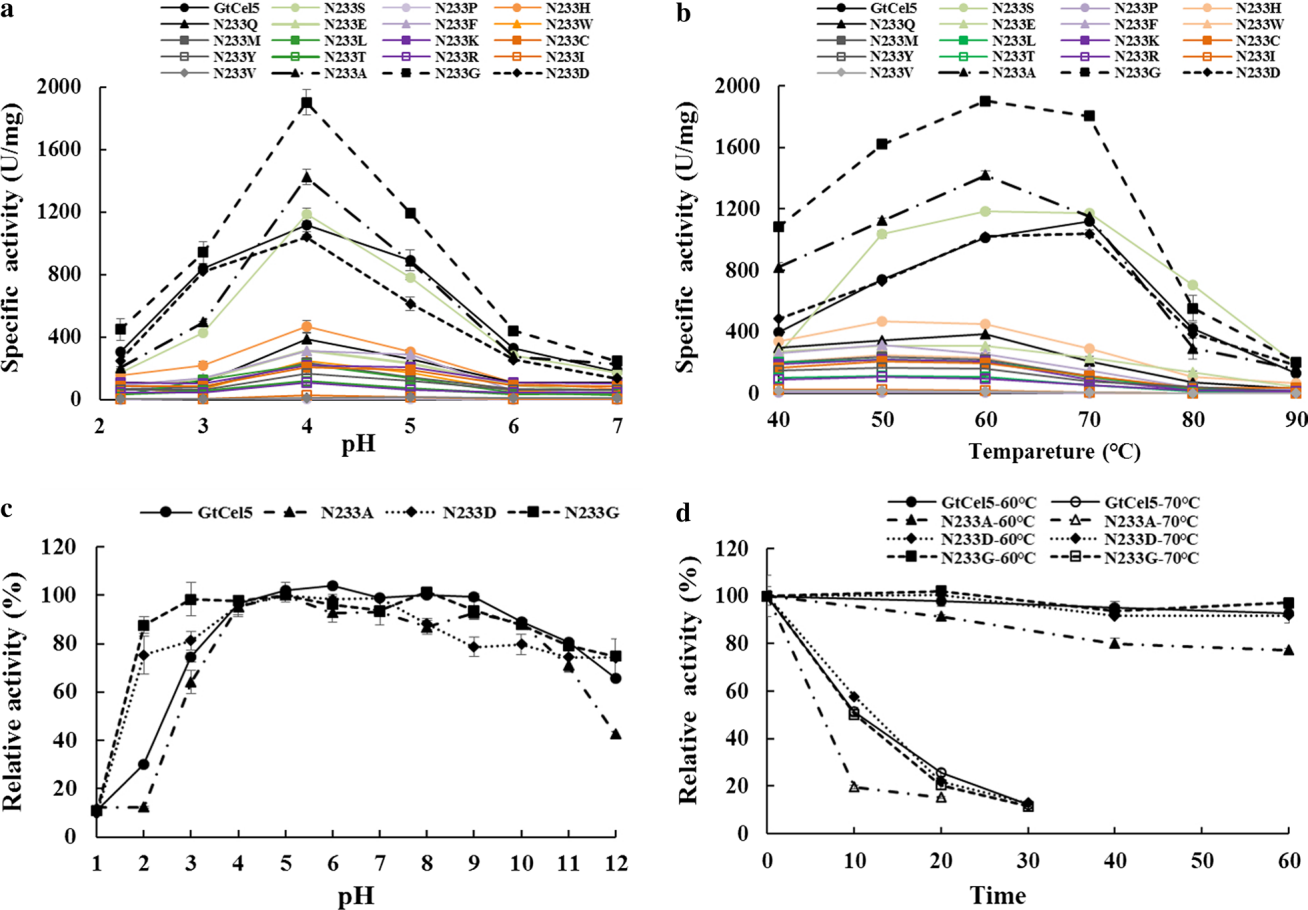
Enzymatic properties of the wild-type *Gt*Cel5 and its variants. **a** pH–activity profiles tested at the optimal temperature of each enzyme with 10 mg/mL CMC-Na as the substrate in 100 mM citric acid–Na_2_HPO_4_ buffer over the pH range of 2.2–7.0 for 10 min. **b** Temperature–activity profiles tested at the optimal pH of each enzyme in the temperature range of 40–90 °C for 10 min. **c** pH–stability profiles determined by measuring the residual activity at optimal pH (100 mM citric acid–Na_2_HPO_4_) and temperature for 10 min after 1-h incubation at pH 1.0–12.0 and 37 °C without substrate. **d** Temperature–stability profiles investigated by measuring the residual activity at optimal pH and temperature after incubation at different temperatures for various durations

When using CMC-Na as the substrate, *Gt*Cel5A showed the highest activity at pH 4.0 and remained more than 30% active at pH values between 2.2 and 6.0 (Fig. 3a). This pH–activity profile is similar to those of most fungal cellulases. The variants except for N233V showed similar pH optima to the wild type, and the optimal pH of N233V shifted to pH 5.0 (Fig. 3a). As shown in Fig. 3b, *Gt*Cel5A had an optimal temperature of 70 °C. All of the variants except for N233D were optimally active at 50 or 60 °C, which was 10–20 °C lower than the wild type, while N233D had similar optimal temperature to the wild type. In comparison with the wild type, all variants showed decreased activities except for N233A, N233G, N233S, and N233D (Fig. 3a, b). The stabilities of *Gt*Cel5A and mutants N233A, N233G, and N233D were also compared. For pH stability, *Gt*Cel5 retained more than 65% of its initial activity after 60-min incubation at 37 °C over a wide pH range (3.0–12.0), while the variants N233D and N233G retained stability over a wider pH range (over 70% activity after 1-h incubation at pH 2.0–12.0) (Fig. 3c). The good stability under both acidic and alkaline conditions makes variants N233D and N233G more favorable for applications in the industries of bioethanol, detergents, and feed additives. *Gt*Cel5 and variants N233A, N233D, and N233G showed similar thermostability (Fig. 3d). The results suggested that the single mutation at position 233 had significant effects on some enzyme properties of *Gt*Cel5.

### Substrate specificities and kinetics of *Gt*Cel5 and its mutants

Of the nine polysaccharide substrates tested, *Gt*Cel5 showed the highest activity on barley β-glucan (6257 ± 26 U/mg) and lichenan (5318 ± 54 U/mg), moderate on CMC-Na (1117 ± 43 U/mg), low toward locust bean gum, and no detectable activity on Avicel, filter paper, xylan, and laminarin. These results indicated that *Gt*Cel5 had no activity on crystalline cellulose and β-1,3 glycosidic linkages. Using CMC-Na as the substrate, *Gt*Cel5 had the *K*_m_, *V*_max_, and *k*_cat_ values of 4.5 ± 0.3 mg/mL, 1475 ± 71 μmol/min/mg, and 878 ± 44/s, respectively, according to the Lineweaver–Burk plot.

CMC-Na was selected as the substrate to compare the specific activities and kinetic values of *Gt*Cel5 and its variants (Table 1). For the substitutions at position 233, the main outcome was a significant decrease in specific activity (1.4–138.6-fold) and *k*_cat_ (0.7–66.5-fold). A few of the variants also showed an increase of *K*_m_ (variants N233M, N233L, N233Y, N233W, N233K, N233V, and N233P). Interestingly, some of the variants gave an increased specific activity, *k*_cat_, and *k*_cat_/*K*_m_ (catalytic efficiency). These were N233A and N233G. These two variants also showed increased specific activities of 1.3- and 1.7-folds toward barley β-glucan in comparison to the wild type (Table 2). The results in combination indicated that glycine or alanine at position 233 contributed to the improved catalytic performance of *Gt*Cel5.

**Table 1:** The kinetic values of *Gt*Cel5, *Te*Egl5A, *Po*Cel5, and their mutants with CMC-Na as the substrate

Enzymes	Specific activity (U/mg)	*K*_m_ (mg/mL)	*V*_max_ (μmol/min/mg)	*k*_cat_ (/s)	*k*_cat_/*K*_m_ (mL/s/mg)
*Gt*Cel5	1117 ± 43	4.5 ± 0.3	1475 ± 71	878 ± 44	195 ± 6
N233G	1901 ± 21	3.6 ± 0.4	1802 ± 96	1072 ± 63	297 ± 8
N233A	1419 ± 22	3.7 ± 0.3	1742 ± 96	1036 ± 57	283 ± 4
N233D	1185 ± 35	4.4 ± 0.7	1511 ± 98	899 ± 87	202 ± 5
N233S	1039 ± 26	7.5 ± 0.2	2302 ± 112	1370 ± 74	184 ± 3
*Te*Egl5A	732 ± 10	7.4 ± 0.3	1014 ± 54	600 ± 24	81.1 ± 7.5
*Te*Egl5A_G216A	523 ± 30	10.9 ± 0.4	1255 ± 94	732 ± 43	61.2 ± 6.7
*Te*Egl5A_G216N	368 ± 15	11.3 ± 0.6	958 ± 76	559 ± 33	49.4 ± 4.3
*Po*Cel5	401 ± 3	5.6 ± 0.3	534 ± 28	311 ± 37	55.1 ± 5.2
*Po*Cel5_G210A	374 ± 2	5.5 ± 0.4	599 ± 36	337 ± 43	62.5 ± 12.1
*Po*Cel5_G210N	166 ± 4	6.9 ± 0.6	280 ± 32	163 ± 24	23. 7 ± 3.2

**Table 2:** The substrate specificities of *Gt*Cel5 and its variants

Enzymes	CMC-Na (U/mg)	Barley β-glucan (U/mg)
*Gt*Cel5	1117 ± 43	6257 ± 26
N233G	1901 ± 21	8383 ± 57
N233A	1419 ± 22	7866 ± 56
N233D	1185 ± 35	5869 ± 33
N233S	1039 ± 26	5474 ± 43

### Reverse mutations on *Te*Egl5A and *Po*Cel5

In order to validate the effect of position 233 on catalytic efficiency, reverse mutation was performed on another two GH5 cellulases: *Te*Egl5A [20] and *Po*Cel5. The corresponding Gly216 of *Te*Egl5A and Gly210 of *Po*Cel5 were substituted by asparagine or alanine, respectively, to generate four variants *Te*Egl5A_G216A, *Te*Egl5A_G216N, *Po*Cel5_G210A, and *Po*Cel5_G210N. All enzymes were successfully produced in *P. pastoris* GS115 and showed bands of theoretical molecular masses after *Endo* H treatment (Additional file 2: Fig. S3).

With CMC-Na as the substrate, *Te*Egl5A and its variants *Te*Egl5A_G216A and *Te*Egl5A_G216N were optimally active at pH 4.0 and 90 °C, while *Po*Cel5 and its variants *Po*Cel5_G210A and *Po*Cel5_G210N showed optimal activities at pH 5.0 and 60 °C (Additional file 2: Fig. S4). These results indicated that the single specific mutation of glycine with asparagine or alanine has no effect on the pH–activity and temperature–activity profiles of *Te*Egl5A and *Po*Cel5. However, great changes were detected on the catalytic performance of variants, as the specific activities of *Te*Egl5A_G216N and *Po*Cel5_G210N decreased to 50 and 41% of the wild types (Table 1). The kinetic values of the variants showed similar trends, i.e., decreased or similar substrate affinity and catalytic efficiencies. The results suggested that glycine at position 233 on loop 6 does make a contribution to the catalytic performance of GH5 cellulases.

### Homology modeling and MD simulation

**Fig. 4:**
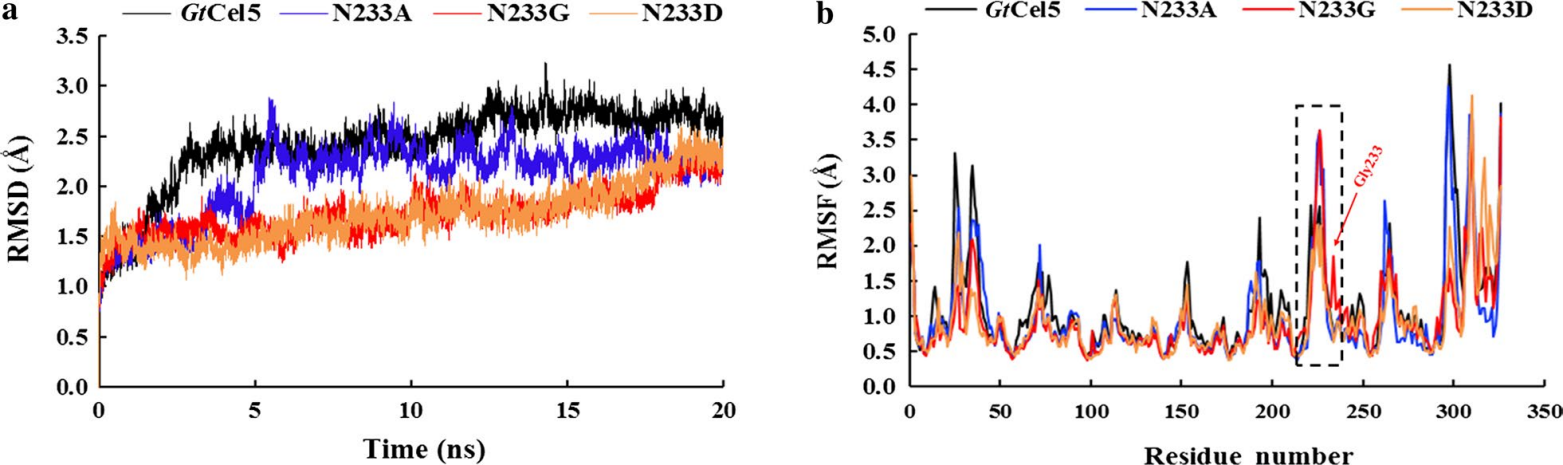
MD simulation analysis of the wild-type *Gt*Cel5 and its variants, N233A, N233D, and N233G, using the force field of AMBER99SB. The data are collected at 323K for a minimum of 20 ns. **a** The RMSD values. The trajectories of first 5 ns were treated as equilibration periods, and the trajectories of the last 15 ns were used for data analyses. **b** The RMSF values. The loop 6 region is indicated by the dashed box

To determine the structural changes caused by mutation at position 233, the modeled structures of *Gt*Cel5 and its variants N233A, N233D, and N233G with and without substrate (G4) were constructed. MD simulations of 20 ns at 323K were then performed. The RMSD of C_α_ atoms tended to be at equilibrium after 5 ns, and thus, the simulation trajectory of the last 5 ns was selected for further analysis. As shown in Fig. 4a, the RMSD values of variants N233A and N233G were lower than that of *Gt*Cel5, suggesting that variants have more stable conformations than the wild type. Moreover, the average RMSF values of N233A (1.84 Å) and N233G (1.87 Å) at loop 6 were higher than that of *Gt*Cel5 (1.74 Å) and N233D (1.46 Å) (Fig. 4b). These results suggested that loop 6, containing the position 233 is more flexible in variants N233A and N233G than in the *Gt*Cel5 and mutant N233D, which may affect the interaction between enzyme and substrate.

**Fig. 5:**
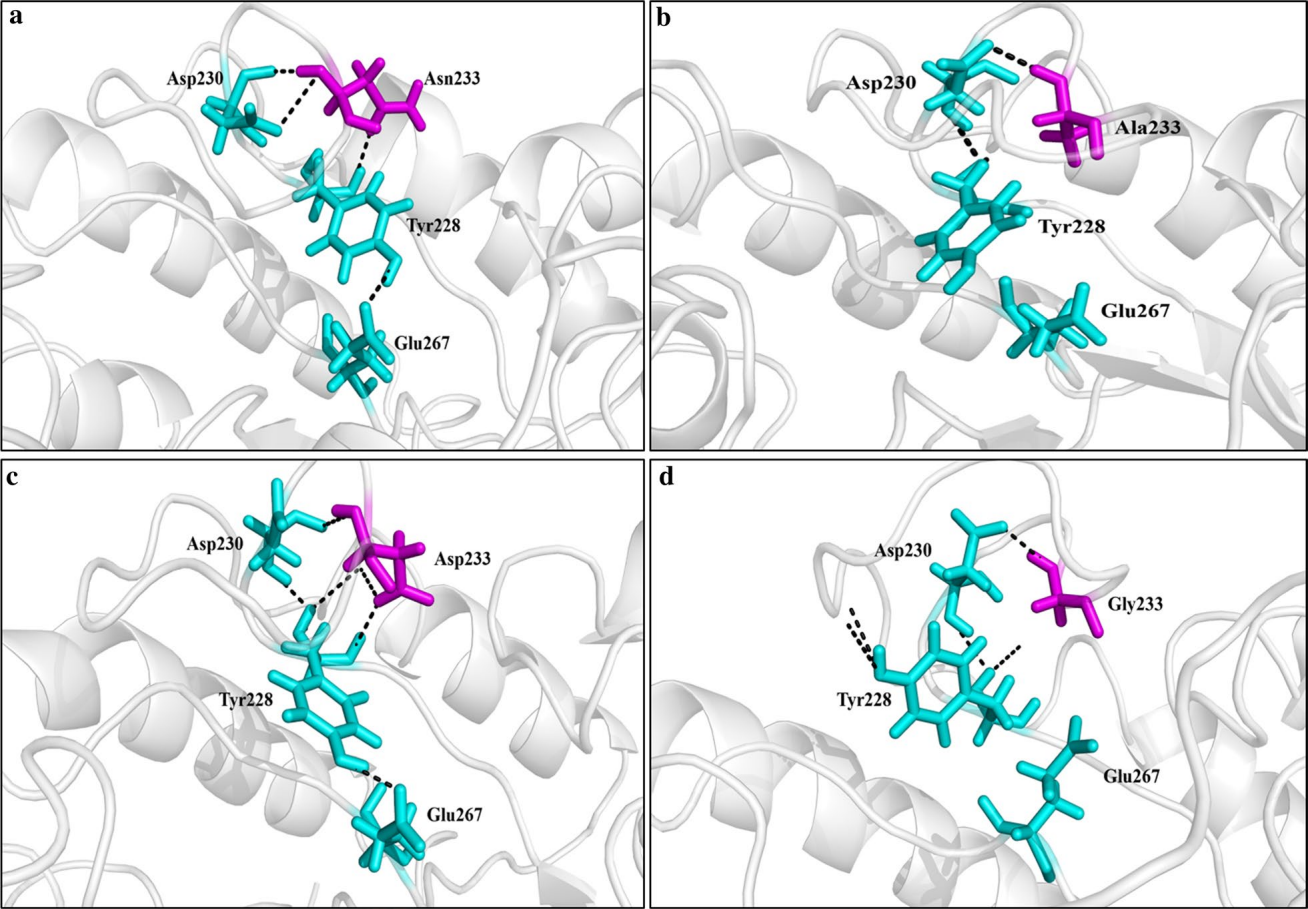
Hydrogen bonds probably formed at position 233 within *Gt*Cel5 and its variants without substrate. The residue of position 233 is shown in purple. **a** *Gt*Cel5. **b** N233A. **c** N233D. **d** N233G

The conformations of *Gt*Cel5 and its variants in the force field AMBER99SB were chosen for the analysis of putative hydrogen bonds. As shown in Fig. 5 and Table 3, the Asn233 of *Gt*Cel5 and the Asp233 of N233D formed two hydrogen bonds with Tyr228 and Asp230, and the Tyr228 formed one more hydrogen bond with the catalytic residue Glu267. These three hydrogen bonds had the occupancy rates of 20–40%. However, in variants N233A and N233G, Ala233, and Gly233 form only one hydrogen bond with Asp230; the occupancy rates were 35 and 53%, respectively; and the hydrogen bond between Tyr228 and Glu267 was absent. The results confirmed that mutation at position 233 has significant effects on the local hydrogen-bonding network.

**Table 3:** Comparison of the hydrogen bond occupancy rates of *Gt*Cel5 and its mutants, N233A and N233G, during the last 15 ns trajectories

Residue^a^	*Gt*Cel5			N233D			N233A			N233G		
	Donor	Acceptor	Occupancy rate (%)	Donor	Acceptor	Occupancy rate (%)	Donor	Acceptor	Occupancy rate (%)	Donor	Acceptor	Occupancy rate (%)
*228/233*	*Y228@H*	*N233@OD1*	*32*	*Y228@H*	*D233@OD2*	*77*	–	–	–	–	–	–
230/233	N233@H	D230@O	20	D233@H	D230@O	13	A233@H	D230@OD2	53	G233@H	D230@OD1	35
*228/267*	*Y228@HH*	*E267@OE2*	*40*	*Y228@HH*	*E267@OE1*	*36*	–	–	–	–	–	–

^a^The hydrogen bonds around position 233 that exist only in wild-type *Gt*Cel5 and N233D are indicated in italics

### Interactions between residue 233 and the substrate

**Fig. 6:**
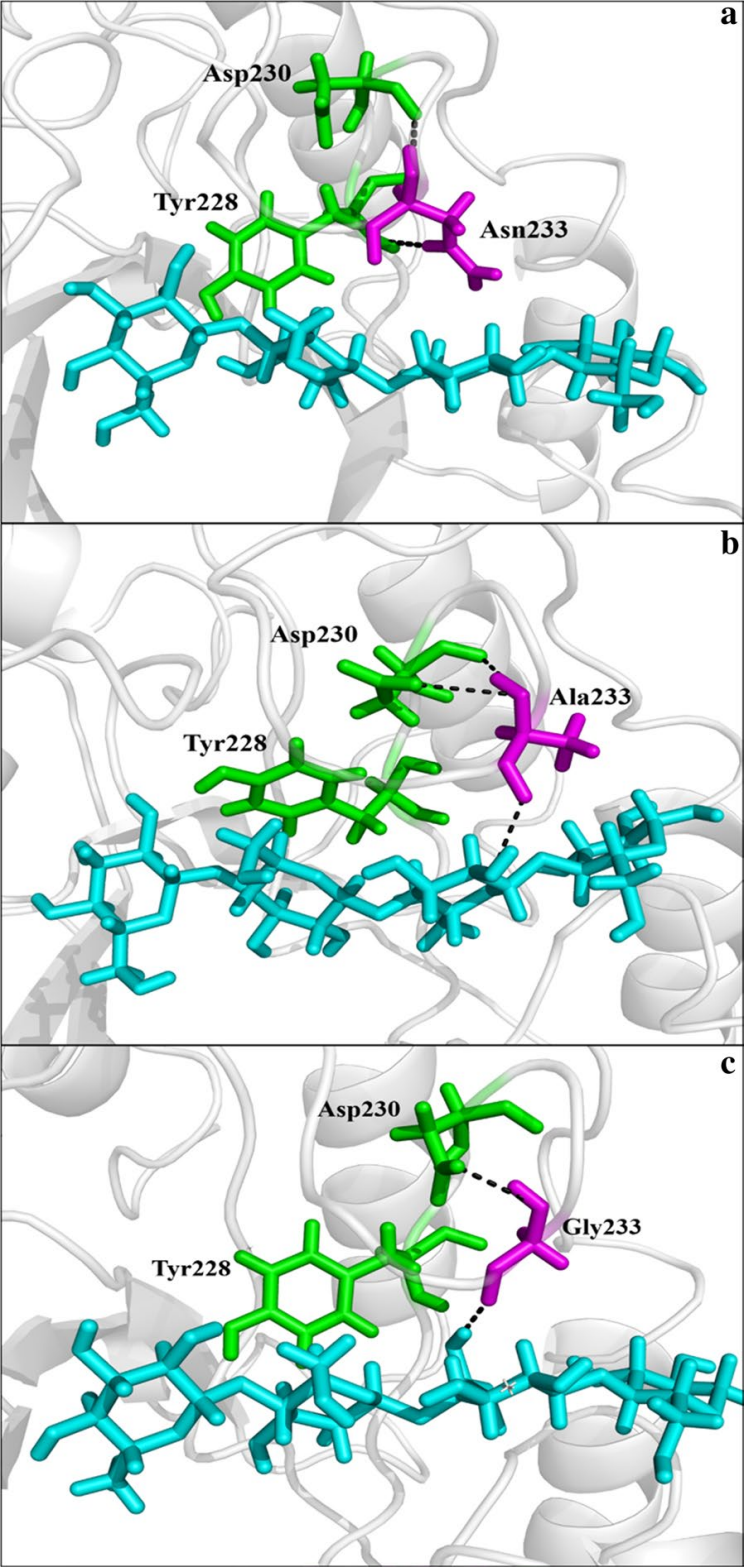
Hydrogen bonds probably formed at position 233 within the complexes of *Gt*Cel5 or its variants and cellotetraose. The residue of position 233 is shown in purple, and the cellotetraose is shown in blue. **a** *Gt*Cel5. **b** N233A. **c** N233G

**Fig. 7:**
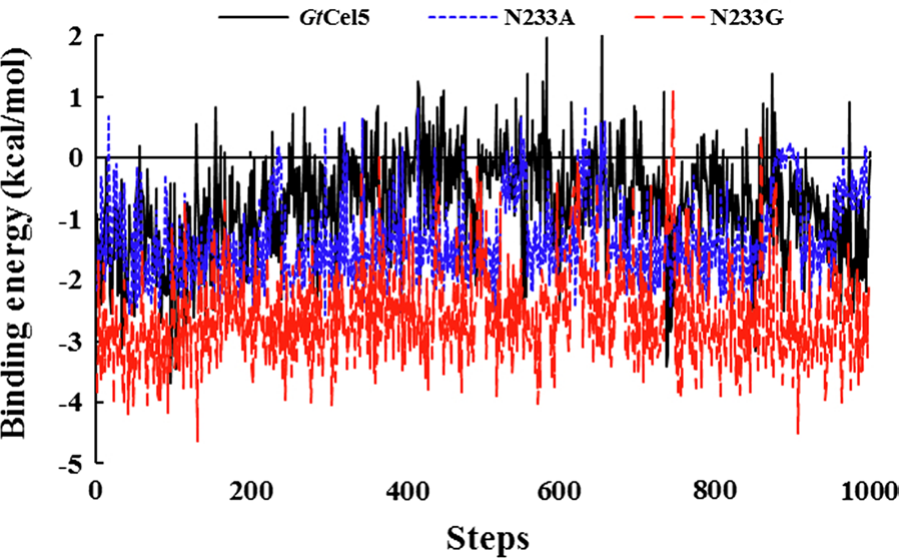
Binding energies of the residue at position 233 within *Gt*Cel5, N233A, and N233G in the last 1000 steps of MD trajectories

The interactions between residue 233 and the substrate were analyzed using the YASARA software. As shown in Fig. 6, one hydrogen bond was formed between the A233@O and G4@H6O in variant N233A–cellotetraose complex or G233@O and G4@H6O in N233G–cellotetraose complex. The occupancy rates of these hydrogen bonds were up to 37 and 45%, respectively. However, this hydrogen bond was absent in the *Gt*Cel5. These results are in accordance with the increased catalytic efficiencies of the two variants. Based on the MM/PBSA calculations, the wild-type *Gt*Cel5 has a binding free energy (*ΔG*) of − 2.7 ± 0.2 k_cal_/mol, while variants N233A and N233G exhibit much lower *ΔG* values (− 22.2 ± 0.2 and − 32.4 k_cal_/mol, respectively). Moreover, the binding energies of *Gt*Cel5 and its variants at position 233 were also calculated. As shown in Fig. 7, N233G showed lower binding energy than that of N233A and *Gt*Cel5. These findings revealed a stronger interaction between the substrate and N233G.

## Discussion

GH5 is a large GH family containing enzymes with broad substrate specificity and various activities, and those from fungi are generally acidic and mesophilic [4]. In this study, an acidic, mesophilic GH5 cellulase was identified in *G. trabeum* CBS 900.73. Based on the key amino acid residue at position 233 of loop 6, the 51 fungal cellulases of GH5 were classified into two main groups: one with asparagine as shown in *Gt*Cel5 and 3QR3 [7], and the other with glycine, as in cellulases *Te*Egl5A, *Po*Cel5 and 1GZJ [6]. The roles of the residue at position 233 were then revealed in *Gt*Cel5 by saturation mutagenesis, which were further verified by reverse mutation in *Gt*Cel5 homologs *Te*Egl5A and *Po*Cel5.

Mobile surface loops have been found to play key roles in protein functions. For example, the thermostability and activity of cellobiohydrolase *Te*Cel7A were improved by introducing more disulfide bridges to the loop structures [34]. As for the *N*-α-acetyl transferase from *Sulfolobus solfataricus*, changing the residues of the loop region between sheets β_3_ and β_4_ destroyed the hydrogen bond network and caused a decrease of 3–7 °C in the protein melting temperature [35]. In the present study, the residue at position 233 was found to have effects on both thermal adaptation and catalytic efficiency of *Gt*Cel5. The temperature–activity profiles of *Gt*Cel5 and its variants showed great variations. Bioinformatic analysis indicated that the local hydrogen bond network of loop 6 (Fig. 5) varied in the enzymes, which probably contribute to the thermal adaptability.

As the best variants, the catalytic performances of N233A and N233G were compared to that of commercial cellulases. When using CMC-Na as the substrate, the specific activities of the widely used cellulase Cel5A from *Hypocrea jecorina* (*Trichoderma reesei*) [36] and the commercial cellulase from *Thermotoga maritime* (Magazyme) are 215.6 and 245 U/mg, respectively, which were much lower than those of N233A (1419 U/mg) and N233G (1901 U/mg). However, other variants had similar or decreased activities (Fig. 3). MD simulation analyses indicated that variants N233G and N233A have higher RMSF values in the region of loop 6, which are correspondent to the improved loop flexibility, especially in N233G. Glycine without side chain has been found to contribute to conformational flexibility of some loop regions, and consequently has effects on enzymatic catalysis and substrate binding. For example, a glycine-rich loop is postulated to undergo conformational change for substrate binding in the mitochondrial-processing peptidase [36], while residue G76 contributes to the active-site loop flexibility of a pepsin [37]. Variants N233A and N233G with more flexible loop 6 showed improvements in substrate affinity (decreased *K*_m_ values), turnover rate (increased *k*_cat_ values), and catalytic efficiency (increased *k*_cat_/*K*_m_ values) (Table 1), which confirmed the effects of alanine and glycine on the loop conformation. Moreover, MD calculation indicated that the improved flexibility of the loop 6 probably affects the hydrogen-bonding network near the active site indirectly (Fig. 5, Table 3). As a result, the conformational freedom of catalytic Glu267 is reduced. However, without the steric hindrance caused by the hydrogen bond between Tyr228 and Glu267, variants N233A and N233G probably experienced a conformational change of the catalytic pocket. Consequently, these variants having higher mobility at loop 6 and a different hydrogen bond pattern at the active site may bind substrates more easily and thus catalyze the hydrolysis of substrate more efficiently.

To the best of our knowledge, hydrogen bonds are also crucial in substrate recognition and binding [38, 39]. Therefore, we also investigated the hydrogen bonds between the enzyme and substrate. MD analysis of the enzyme–substrate complex dynamics indicated that the Asn233 of *Gt*Cel5 has no direct ligand contact with G4, while Ala233 or Gly233 of variants N233A and N233G was more likely to form a hydrogen bond with G4 with higher occupancy rates. Although this hydrogen bond was also identified in N233D, the occupancy rate was much lower (27%). This result is in agreement with the increased catalytic efficiencies of variants N233A and N233G. Similar results have been reported in the *Tr*Cel7A from *T. reesei*, in which hydrogen bond interaction exists in the whole catalytic process and plays a role of special importance in stabilizing the intermediate state and improvement of the catalytic performance [40]. Besides, in *Tl*Xyn10A_P from *Talaromyces leycettanus*, G149D on the loop 4 is able to form a hydrogen bond with substrate and probably plays a major role in the improvement of catalytic performance [41]. To analyze the binding affinity of substrate and enzyme, we performed MM/PBSA calculations, and found that the binding energies of *Gt*Cel5 and its variants are in the order of N233G < N233A < *Gt*Cel5. These data are correspondent to the experimental work that showed N233G and N233A having higher affinity with cellotetraose than with*Gt*Cel5 (Tables 1, 2). Therefore, the substitution of Asn233 with alanine or glycine might cause the enzyme to form more stable hydrogen bonds with the substrate and improve the interactions between enzyme and substrate, and consequently enhance the substrate’s binding and catalytic efficiencies.

## Conclusions

In the present study, an acidic, mesophilic cellulase of GH5 was identified in *G. trabeum* CBS 900.73 and produced in *P. pastoris* GS115. Structure and sequence analyses indicated that the residue at position 233 on loop 6 plays a crucial role in the catalytic performance of GH5 cellulases. By increasing the local hydrogen bond interactions around the residue at position 233 and between the enzyme and substrate, the substrate affinity was enhanced, as was the catalytic efficiency. Considering the significance of GH5 cellulases in biomass conversion, the findings are valuable for the protein engineering of GH5 cellulases in the viewpoints of research, development, and industrial applications.

## Additional files

**Additional file 1: Table S1.** Primers used in this study.

**Additional file 2: Fig. S1.** The phylogenetic analysis of *Gt*Cel5 and other GH5 enzymes of bacterial and fungal sources. GenBank accession numbers or PDB numbers are shown. **Fig. S2.** SDS-PAGE analysis of *Gt*Cel5 and its variants. M, the molecular weight markers; 1, 4, 7 and 10, the crude enzymes; 2, 5, 8 and 11, the purified enzymes; 3, 6, 9 and 12, the deglycosylated enzymes with *Endo* H treatment. **Fig. S3.** SDS-PAGE analysis of *Te*Egl5A, *Po*Cel5 and their variants. M, the molecular weight markers; 1, 4 and 7, the crude enzymes; 2, 5 and 8, the purified enzymes; 3, 6 and 9, the deglycosylated enzymes with *Endo* H treatment. **Fig. S4.** Enzymatic properties of the wild type *Te*Egl5A, *Po*Cel5 and their variants. (A) pH-activity profiles of *Te*Egl5A and its variants tested at the optimal temperature of each enzyme (90 °C) over the pH range of 3.0–7.0 for 10 min. (B) Temperature-activity profiles of *Te*Egl5A and its variants tested at the optimal pH of each enzyme in the temperature range of 50–95 °C for 10 min. (C) pH-activity profiles of *Po*Cel5 and its variants tested at the optimal temperature (60 °C) over the pH range of 3.0–8.0 for 10 min. (D) Temperature-activity profiles of *Po*Cel5 and its variants tested at the optimal pH of each enzyme in the temperature range of 40–90 °C for 10 min.

## Abbreviations

CMC-Na: carboxymethycellulose sodium
G4: cellotetraose
MD: molecular dynamics
RMSD: root-mean-square deviation
RMSF: root-mean-square fluctuation
MM/PBSA: molecular mechanics/Poisson Boltzmann and surface area continuum solvation
