# Supplementary material for: Enhancing the catalytic activity of a novel GH5 cellulase GtCel5 from Gloeophyllum trabeum CBS 900.73 by site-directed mutagenesis on loop 6
Source: Biotechnol Biofuels. 2018 Mar 22;11:76. doi: 10.1186/s13068-018-1080-5 (PMC5863444; doi:10.1186/s13068-018-1080-5)
Supplement: Supplementary file 1 — Additional file 1: Table S1. Primers used in this study. [file 13068_2018_1080_MOESM1_ESM.docx]

**Additional files to**

**Enhancing the catalytic activity of a novel GH5 cellulase *Gt*Cel5 from *Gloeophyllum trabeum* CBS 900.73 by site-directed mutagenesis on loop 6**

Fei Zheng^1,2^, Tao Tu^1^, Xiaoyu Wang^2^, Yuan Wang^1^, Rui Ma^1^, Xiaoyun Su^1^, Xiangming Xie^2^, Bin Yao^1*^ and Huiying Luo^1*^

^1^ Key Laboratory for Feed Biotechnology of the Ministry of Agriculture, Feed Research Institute, Chinese Academy of Agricultural Sciences, Beijing 100081, People’s Republic of China.

^2^ College of Biological Sciences and Biotechnology, Beijing Forestry University, Beijing 100083, People’s Republic of China.

**E-mail addresses:**

FZ: zhengfei0718@sina.com

TT: tutao@caas.cn

XW: [showery2011@hotmail.com](mailto:showery2011@hotmail.com)

YW: wangyuan08@caas.cn

RM: [marui@caas.cn](mailto:marui@caas.cn)

XS: suxiaoyun@caas.cn

XX: xxm1005@126.com

BY: binyao@caas.cn

HL: [luohuiying@caas.cn](mailto:luohuiying@caas.cn)

* Corresponding authors.

**Additional file 1: Table S1 Primers used in this study.**

| **Primers** | **Sequences (5′→3′)^a^** |
| --- | --- |
| *Gtcel5-F* | CCGAATTCGCCGCGCTCTCTCCGAGAGTGACA |
| *Gtcel5-R* | TAGCGGCCGCTCATGCGTTGGCAATCGGAGCCAAGCA |
| *P1* | ACTTGGACTCCGACNNKTCCGGCACGAG |
| *P2* | CTCGTGCCGGAMNNGTCGGAGTCCAAGT |
| *TeEgl5A_G216A-F* | ACCTGGACTCGGACGCGTCGGGCACG |
| *TeEgl5A_G216A-R* | GCGTCCGAGTCCAGGTACTGGTGCA |
| *TeEgl5A_G216N-F* | TACCTGGACTCGGACAACTCGGGCACGT |
| *TeEgl5A_G216N-R* | TTGTCCGAGTCCAGGTACTGGTGCAT |
| *PoCel5_G210A-F* | ACCTCGATGTAGACGCCAGCGGTACG |
| *PoCel5_G210A-R* | GCGTCTACATCGAGGTATTGATGCA |
| *PoCel5_G210N-F* | TACCTCGATGTAGACAACAGCGGTACG |
| *PoCel5_G210N-R* | TTGTCTACATCGAGGTATTGATGCAT |
