# Supplementary material for: Enhancing the catalytic activity of a novel GH5 cellulase GtCel5 from Gloeophyllum trabeum CBS 900.73 by site-directed mutagenesis on loop 6
Source: Biotechnol Biofuels. 2018 Mar 22;11:76. doi: 10.1186/s13068-018-1080-5 (PMC5863444; doi:10.1186/s13068-018-1080-5)
Supplement: Supplementary file 2 — Additional file 2: Fig. S1. The phylogenetic analysis of GtCel5 and other GH5 enzymes of bacterial and fungal sources. GenBank accession numbers or PDB numbers are shown. Fig. S2. SDS-PAGE analysis of GtCel5 and its variants. M, the molecular weight markers; 1, 4, 7 and 10, the crude enzymes; 2, 5, 8 and 11, the purified enzymes; 3, 6, 9 and 12, the deglycosylated enzymes with Endo H treatment. Fig. S3. SDS-PAGE analysis of TeEgl5A, PoCel5 and their variants. M, the molecular weight markers; 1, 4 and 7, the crude enzymes; 2, 5 and 8, the purified enzymes; 3, 6 and 9, the deglycosylated enzymes with Endo H treatment. Fig. S4. Enzymatic properties of the wild type TeEgl5A, PoCel5 and their variants. (A) pH-activity profiles of TeEgl5A and its variants tested at the optimal temperature of each enzyme (90 °C) over the pH range of 3.0–7.0 for 10 min. (B) Temperature-activity profiles of TeEgl5A and its variants tested at the optimal pH of each enzyme in the temperature range of 50–95 °C for 10 min. (C) pH-activity profiles of PoCel5 and its variants tested at the optimal temperature (60 °C) over the pH range of 3.0–8.0 for 10 min. (D) Temperature-activity profiles of PoCel5 and its variants tested at the optimal pH of each enzyme in the temperature range of 40–90 °C for 10 min. [file 13068_2018_1080_MOESM2_ESM.doc]

**Additional files to**

**Enhancing the catalytic activity of a novel GH5 cellulase *Gt*Cel5 from *Gloeophyllum trabeum* CBS 900.73 by site-directed mutagenesis on loop 6**

Fei Zheng^1,2^, Tao Tu^1^, Xiaoyu Wang^2^, Yuan Wang^1^, Rui Ma^1^, Xiaoyun Su^1^, Xiangming Xie^2^, Bin Yao^1*^ and Huiying Luo^1*^

^1^ Key Laboratory for Feed Biotechnology of the Ministry of Agriculture, Feed Research Institute, Chinese Academy of Agricultural Sciences, Beijing 100081, People’s Republic of China.

^2^ College of Biological Sciences and Biotechnology, Beijing Forestry University, Beijing 100083, People’s Republic of China.

**E-mail addresses:**

FZ: zhengfei0718@sina.com

TT: tutao@caas.cn

XW: [showery2011@hotmail.com](mailto:showery2011@hotmail.com)

YW: wangyuan08@caas.cn

RM: [marui@caas.cn](mailto:marui@caas.cn)

XS: suxiaoyun@caas.cn

XX: xxm1005@126.com

BY: binyao@caas.cn

HL: [luohuiying@caas.cn](mailto:luohuiying@caas.cn)

* Corresponding authors.

**Additional file 2:**


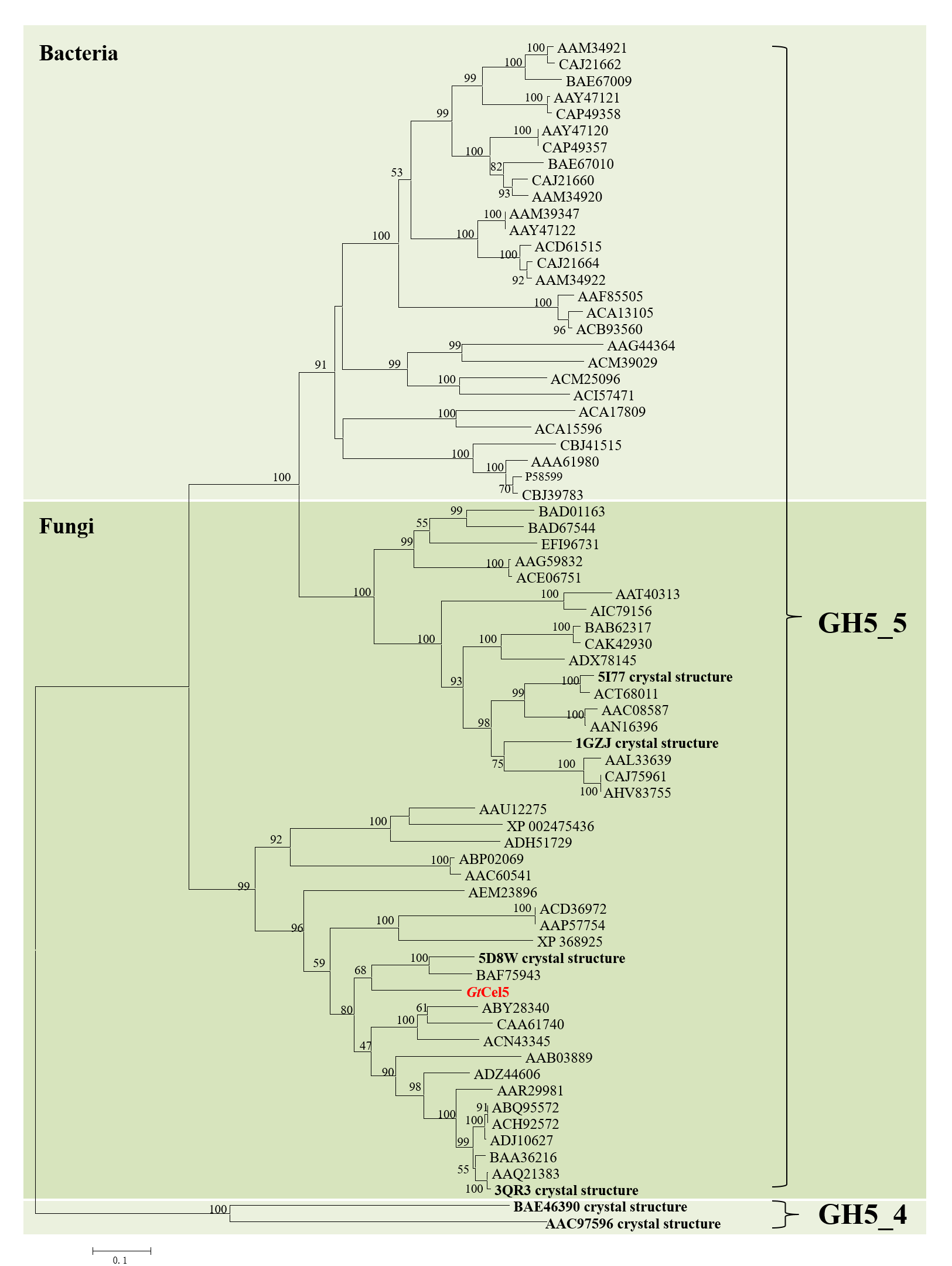


**Fig. S1. The phylogenetic analysis of *Gt*Cel5 and other GH5 enzymes of bacterial and fungal sources.** GenBank accession numbers or PDB numbers are shown.

**
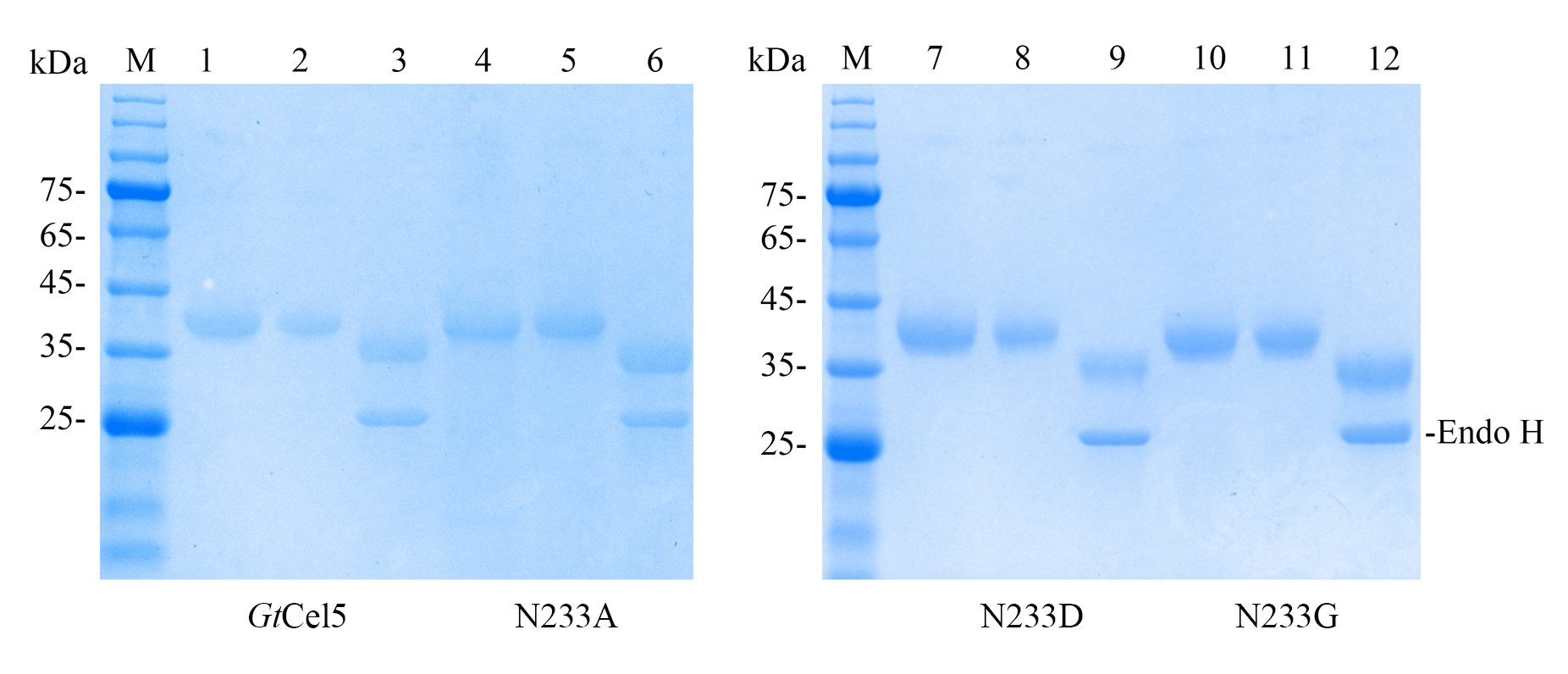
**

**Fig. S2. SDS-PAGE analysis of *Gt*Cel5 and its variants.** M, the molecular weight markers; 1, 4, 7 and 10, the crude enzymes; 2, 5, 8 and 11, the purified enzymes; 3, 6, 9 and 12, the deglycosylated enzymes with Endo H treatment.


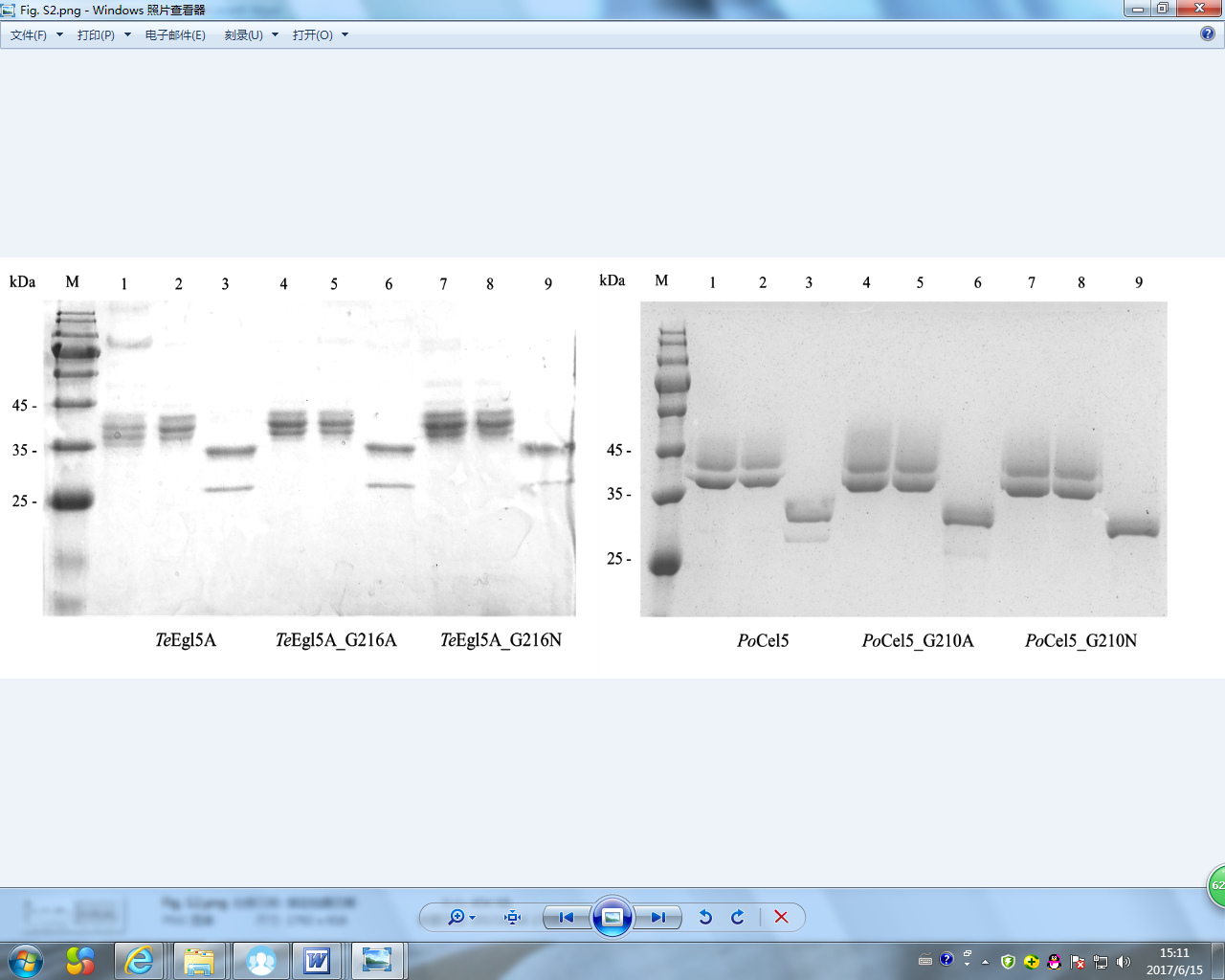


**Fig. S3. SDS-PAGE analysis of *Te*Egl5A, *Po*Cel5 and their variants.** M, the molecular weight markers; 1, 4 and 7, the crude enzymes; 2, 5 and 8, the purified enzymes; 3, 6 and 9, the deglycosylated enzymes with Endo H treatment.

**
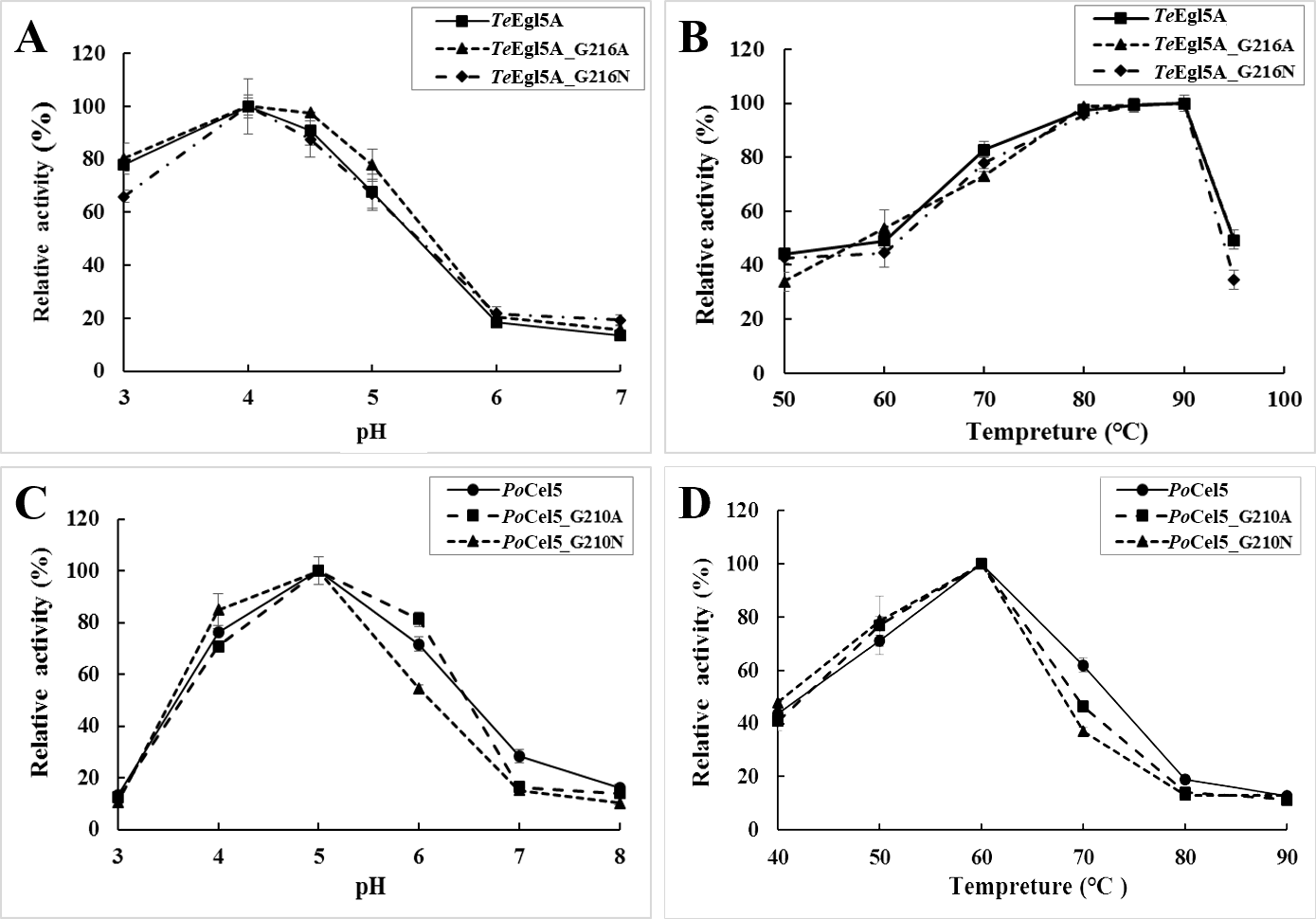
**

**Fig. S4. Enzymatic properties of the wild type *Te*Egl5A, *Po*Cel5 and their variants.** (A) pH-activity profiles of *Te*Egl5A and its variants tested at the optimal temperature of each enzyme (90 °C) over the pH range of 3.0–7.0 for 10 min. (B) Temperature-activity profiles of *Te*Egl5A and its variants tested at the optimal pH of each enzyme in the temperature range of 50–95 °C for 10 min. (C) pH-activity profiles of *Po*Cel5 and its variants tested at the optimal temperature (60 °C) over the pH range of 3.0–8.0 for 10 min. (D) Temperature-activity profiles of *Po*Cel5 and its variants tested at the optimal pH of each enzyme in the temperature range of 40–90 °C for 10 min.
